# Color Cherenkov imaging of clinical radiation therapy

**DOI:** 10.1038/s41377-021-00660-0

**Published:** 2021-11-04

**Authors:** Daniel A. Alexander, Anthony Nomezine, Lesley A. Jarvis, David J. Gladstone, Brian W. Pogue, Petr Bruza

**Affiliations:** 1 Thayer School of Engineering, Dartmouth College, Hanover, NH USA; 2 DoseOptics LLC, Lebanon, NH USA; 3 Geisel School of Medicine, Dartmouth College, Hanover, NH USA; 4 Norris Cotton Cancer Center, Dartmouth-Hitchcock Medical Center, Lebanon, NH USA

**Keywords:** Imaging and sensing, Optical sensors

## Abstract

Color vision is used throughout medicine to interpret the health and status of tissue. Ionizing radiation used in radiation therapy produces broadband white light inside tissue through the Cherenkov effect, and this light is attenuated by tissue features as it leaves the body. In this study, a novel time-gated three-channel camera was developed for the first time and was used to image color Cherenkov emission coming from patients during treatment. The spectral content was interpreted by comparison with imaging calibrated tissue phantoms. Color shades of Cherenkov emission in radiotherapy can be used to interpret tissue blood volume, oxygen saturation and major vessels within the body.

## Abstract (web summary)

A novel three-channel intensified camera capable of imaging Cherenkov emission in full color was developed, allowing for sensitivity to in vivo biological tissue information during radiotherapy.

## Graphical abstract

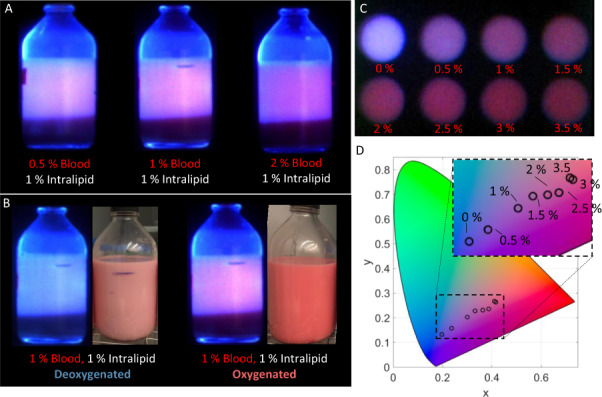

## Introduction

Color vision has been perhaps the most widely used diagnostic in medicine, being part of every point-of-care examination. The human eye can perceive millions of color shades^1^, making it one of the best diagnostic devices that exist, and physicians take advantage of this every day. In this work, the concept of color imaging of radiotherapy dose delivery via the Cherenkov effect has been developed and demonstrated in clinical radiation therapy. The attenuation and transmission of this light through human tissues alter the perceived color signal emitted from the patient’s surface, thereby yielding biological tissue information. While Cherenkov emission has been known to exist for many decades^2,3^, this is the first in-depth examination of what could be gained from multiwavelength imaging of this phenomenon in the context of radiation therapy.

**Fig. 1:**
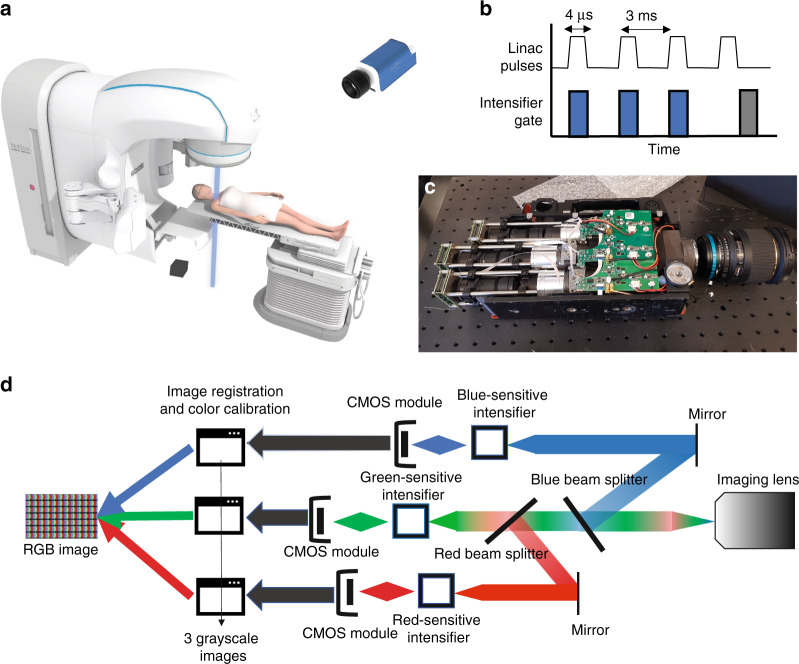
Imaging scheme and camera design. Time-resolved imaging of Cherenkov emission from patients, as shown in **a**, is possible because the radiation is delivered in 4 ms pulses at a very low duty cycle, and time-gated intensified cameras can therefore capture the Cherenkov signal and perform real-time background subtraction (**b**). Performing color Cherenkov imaging requires time-gating in each color channel, necessitating the inclusion of three sets of imaging hardware and appropriate beam splitting optics behind the imaging lens, which is realized in **c**. A detailed schematic of the custom three-channel camera is highlighted in **d**

X-rays are widely used to image through tissue, however, until recently, it has not been possible to directly visualize their interaction within tissue during dose deposition. Theory and simulations predict a linear relationship between Cherenkov emission and radiation dose under specific conditions^4^, and therefore Cherenkov light imaging has provided a tool to image the dose delivery from high energy X-rays. Time-gated imaging of Cherenkov emission is able to record the dose delivery process in real-time and visualize radiotherapy treatments from start to finish^5,6^. The ability to view the beam incidence upon tissue provides an intuitive sense of how to ensure that daily treatments are being delivered with sufficient accuracy^7,8^. To date, the systems developed for Cherenkov imaging dosimetry have been monochrome intensified cameras, and due to the red and near-infrared weighted emission from tissue^9,10^, little focus has been given to in vivo Cherenkov imaging in other wavelength bands. However, changes in blood content and oxygenation within a tissue have large effects upon the light emission, and the spectral changes associated with these are known to be diagnostic. In this study, a three-sensor camera was prototyped with a time-gated image intensifier on each channel to image the color of Cherenkov light emitted during radiation therapy. As radiation therapy is primarily delivered from a linear accelerator with short pulses of radiation, time-gating allowed for detection of this low-intensity signal above background ambient light levels. In addition to the goal of seeing the true color of Cherenkov emission from tissue, this work was initiated to explore the hypothesis that changes in oxygenation and tissue composition could be detected in the images. A detailed schematic of the camera assembly developed for this study is shown in Fig. 1. Following phantom imaging, a systematic color study was performed and accompanied by patient imaging, to assess the value of these colors in characterizing radiotherapy dose delivery.

## Results

### Derived Cherenkov emission spectra from tissue

**Fig. 2:**
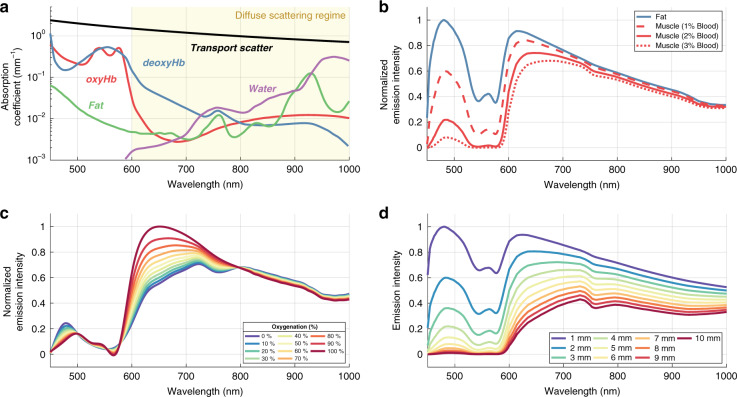
Cherenkov light transport in tissue. The spectrum of light emitted from tissue is predominantly affected by the absorption bands from oxyhemoglobin, deoxyhemoglobin, water, and lipids, and the overall tissue scattering spectrum, shown in **a**. The calculated Cherenkov emission from 5 mm depth in fatty (90% fat/9.5% water/0.5% blood (0.25% Hb/0.25% HbO_2_)) and radiodense (water and blood in varying percentages with equal weight to Hb and HbO_2_) tissues are shown in **b**, whereas biological changes in blood oxygenation in radiodense tissue (2% blood) a will alter the Cherenkov emission spectrum due to differences in absorption between oxy- and deoxyhemoglobin **c**. Lastly, varying the depth of emission in radiodense tissue (2% blood) effects the Cherenkov emission spectrum at the surface (**d**)

Utilizing the available spectra of extinction coefficients for various biological media in the visible and near-infrared range^11–14^ as shown in Fig. 2a, Cherenkov emission spectra were calculated for both fat and muscle tissue types with a fixed emission depth of 5 mm using the following linear combinations of the absorption coefficient for fatty and muscle tissue:

1 $$\begin{array}{l} \mu _{{\rm{Tot}},{\rm{Fatty}}}\left( \lambda \right) = 0.9\mu _{a,{\rm{Water}}}\left( \lambda \right) + 0.095\mu _{a,{\rm{Fat}}}\left( \lambda \right) \\ \qquad \qquad\qquad\;+\, 0.005\mu _{a,{\rm{Blood}}}\left( \lambda \right)\end{array}$$

2 $$\mu _{{\rm{Tot}},{\rm{Muscle}}}\left( \lambda \right) = 0.98\mu _{a,{\rm{Water}}}\left( \lambda \right) + 0.02\mu _{a,{\rm{Blood}}}\left( \lambda \right)$$

These spectra are shown in Fig. 2b, along with variations in the muscle blood content to 1% and 3%, indicating that Cherenkov emission from fatty tissues contains a higher proportion of blue light when compared with that from muscle tissue. Furthermore, by varying the weight of oxyhemoglobin in the combined blood absorption coefficient in Eq. 2, the Cherenkov emission spectrum from muscle tissue with varying levels of oxygenation was extracted (Fig. 2c). This result highlights the shift in spectral profile towards red-weighted emission for increasing levels of tissue oxygenation. At last, emission depth was varied from 10 mm to 1 mm for the same muscle tissue type (Fig. 2d), outlining the depth-dependent contributions to the color of Cherenkov emission at the surface of the tissue, with red-weighting increasing monotonically with depth.

### Variation in color of Cherenkov emission from tissue phantoms

**Fig. 3:**
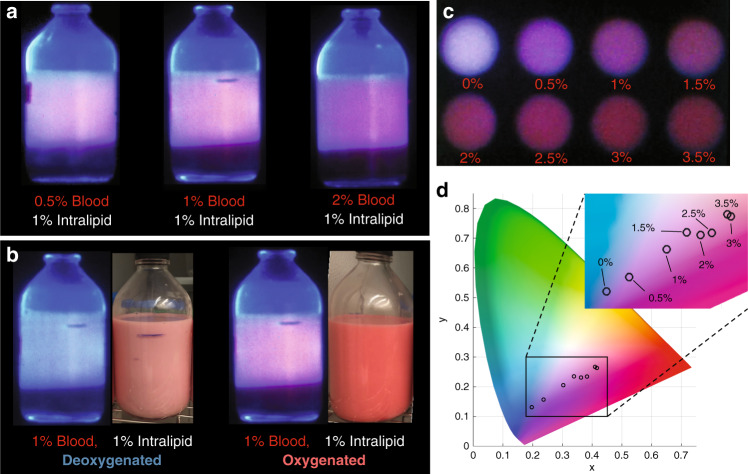
Color change of cherenkov emission with varying tissue parameters. Varying blood concentration in aqueous solutions containing 1% intralipid to mimic tissue scatter impacts the color of Cherenkov emission from the samples, shown in **a** under irradiation with a 6 MV X-ray beam. In addition, increased oxygenation increases the signal in the red channel of the Cherenkov images shown in **b** due to the decreased absorption of red light by oxyhemoglobin compared with deoxyhemoglobin. Systematic variation of the blood concentration in small samples of 1% intralipid solution in **c** shows a near-monotonic distribution of colors along the red-to-blue axis when overlaid on a CIE chromaticity diagram in the xyY color space (**d**)

White light images of a tissue phantom with oxygenated porcine blood at 1% show an increased red hue, when compared with 1%, deoxygenated blood as shown in Fig. 3b, which is reflected in the color Cherenkov images. Notably present is the blue glow around the outer edge of the phantom contributed by Cherenkov light generated in the glass, which is also reflected in the phantom images with varying blood concentrations (Fig. 3a). Systematic alteration of blood concentration in liquid tissue phantoms yielded an appreciable hue shift from blue towards red as displayed in Fig. 3c, and this effect was quantified by analyzing the average color value in each phantom in the CIE xyY color space overlayed on a chromaticity diagram. This portrayal in Fig. 3d reveals a near-monotonic increase in the derived luminance parameter *x* with increasing blood concentration from 0.5% to 3.5%, and a linear fit to these data yielded a strong linear correlation (*R*^2^ = 0.96). This result agrees with the perceptual observation of increasing blood concentration yielding a redder image, and with the predicted emission spectra from muscle tissue with varying blood concentrations shown in Fig. 2b.

### Coincidence of color features and anatomical landmarks in patient images

**Fig. 4:**
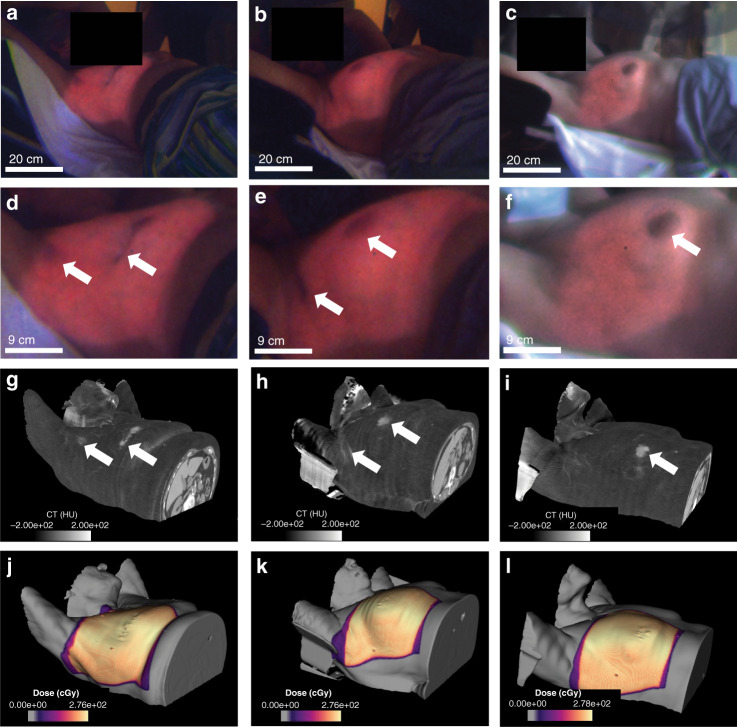
Coincidence of Cherenkov color variations and surface tissue features. Cherenkov images of whole breast radiotherapy treatment of three patients **a**–**c**, with zoomed-in views below **d**–**f**. The outer edge of the radiation field delineated by the Cherenkov emission corresponds with the surface dose distributions extracted from the treatment planning system (**j**–**l**). Surface-weighted tissue density maps extracting from the simulation CT scan (**g**–**i**) show high-density features including the nipple-areolar complex, axillary scars, and surface vasculature, which are coincident with color variations in the corresponding Cherenkov images as highlighted with white arrows

Figure 4 shows color Cherenkov images for three patients who were receiving whole breast radiotherapy subsequent to their surgical resection for breast cancer. These images are shown along with corresponding surface tissue x-ray CT density maps and surface dose maps extracted from the clinical treatment planning software. It can be seen from a qualitative comparison of the color Cherenkov images to the dose maps that the edge of the Cherenkov emission region corresponds to the edge of the surface dose distribution, with the contrast between signal and background in the Cherenkov images provided from color differences between irradiated and spared tissues. In addition, anatomical features readily identifiable on the surface CT map such as the nipple-areolar complex, axillary and periareolar incision scars, and surface vasculature are shown to have reduced red channel intensity in corresponding regions of the color Cherenkov images, highlighting in vivo color variations in Cherenkov emission from different surface-level tissue types, in agreement with derived spectral predictions and phantom observations. As both Cherenkov and the background signal are shown together in color, it is worth noting that certain features such as the nipple are visible both with room-light illumination and from Cherenkov emission. Figure S1 shows the raw images acquired for each patient prior to color correction, highlighting the differences in room lighting between the first two patients (Fig. 4a, b) and the third (Fig. 4c).

## Discussion

This work presents a novel demonstration of three-channel color imaging of Cherenkov emission, both in phantoms and in vivo during patient treatment. Presented in this work is both the prediction and verification that Cherenkov emission spectral characteristics are altered by varying tissues, and this effect manifests as perceptible color changes in a biologically relevant range of parameters. Previous studies have shown that monochrome, red-weighted Cherenkov imaging is able to provide contrast for surface-level anatomical features and is sensitive to tissue density^7,15^, and here it is shown that this concept can be extended to three-channel color images. The images in Fig. 4 show that various tissue features manifest not only with varying intensities, as previously demonstrated, but with unique spectral signatures. Combining spatially resolved color information from Cherenkov images with reflectance- and/or tissue density-based will allow for more accurate Cherenkov-to-dose conversion models for in vivo surface dosimetry^15,16^. This increased sensitivity to these features may also contribute to more accurate day-to-day position tracking of the patient setup, and the novel addition of biological information such as sensitivity to blood volume and oxygenation can inform clinicians about physiologic responses to therapy, such as the development of erythema.

Although this technique does require background light suppression through time-gating to the linac pulses, one advantage is the ability to acquire useful images without background subtraction, as the contrast between Cherenkov signal and background is achieved through color perception. This technique also presents a natural overlay of Cherenkov light and background signals through concurrent acquisition and accumulation, allowing for more realistic visualizations of radiation interaction with treated tissues.

One primary disadvantage of the described method is the complexity of the camera design, requiring three independent intensified CMOS cameras. In addition, image analysis and calibration are more involved when compared with conventional monochrome Cherenkov imaging owing to the complex gamma calibration of the red-green-blue (RGB) image channels.

Future work will include further patient imaging studies, specifically to investigate the impact of inter-patient large-scale tissue composition differences on the color of Cherenkov emission; this would necessitate careful control over camera position and consistency of treatment room lighting. Furthermore, this initial study only involved imaging patients with light skin pigmentation due to the local patient demographics, and it will be necessary to image a broader spectrum of skin pigmentation levels in future studies. In addition, the use of three-channel Cherenkov emission sensing will be investigated as a tool to improve Cherenkov-to-dose correction factors for patient-specific small-scale tissue optical property variations and across larger patient cohorts.

## Materials and methods

### Camera components

Each channel of the color camera is composed of an independent intensified CMOS (iCMOS) camera (C-Dose, DoseOptics LLC, Lebanon NH USA) housed in a three-tube color video camera assembly (JVC, Yokohama, Japan). The red channel was outfitted with a red-sensitive iCMOS camera, whereas the blue and green channels contained blue-sensitive iCMOS cameras. The imaging characteristics and spectral sensitivity of these cameras have been explored previously^17,18^. Each camera was remotely triggered to stray X-rays, allowing for synchronization with and gating to the linear accelerator pulses^19^. The beamsplitter assembly of the video camera, consisting of RGB dichroic beam splitters and bandpass filters, allowed for incoming Cherenkov light signals to be redirected according to wavelength to the appropriate camera channel resulting in three raw image stacks for each acquisition. The camera was equipped with a 10–100 mm, *f*/1.6 zoom lens (JVC, Yokohama, Japan).

### Image acquisition and processing

Images were acquired using the C-Dose Research software (DoseOptics LLC, Lebanon NH USA) in 16-bit raw format. Temporal and spatial median filtering were applied and performed on each camera’s FPGA, with 5-frame and 5 × 5-pixel moving windows, respectively. This step removed the need for additional noise removal or smoothing in post-processing. Darkfield image stacks were acquired for 120 frames with the lens cap applied, and the average darkfield frame was calculated. All post-acquisition image analysis was performed in MATLAB (MathWorks, Natick MA, USA). For a given acquisition dataset, each of the three image stacks was frame-averaged and the average darkfield frame was subtracted from each. Next, using a checkerboard-based geometric calibration, 2D polynomial transformations were applied to the green and blue images to spatially align the three channels, resulting in a spatially registered three-channel image.

### Color calibration

Color calibration was performed using a standardized color chart (Spyder Checkr 24, Datacolor, Lucerne, Switzerland). Images of the color chart were acquired and ROIs for each color panel were delineated. The resulting values for each channel were compared with those from the reference image of the color chart, and a linear fit was applied. These fit coefficients were then used to color correct the raw pixel values in the uncorrected color images for each acquisition.

### Phantom design and imaging

For testing the impact of oxygenation on the color of Cherenkov emission, tissue phantoms were created using aqueous solutions of whole porcine blood (Lampire Biological Labs, Pipersville PA) and intralipid (Baxter Inc., Glenview IL) at 1% each in 1 L glass bottles and glucose oxidase was used to deoxygenate one sample. In addition, phantoms with varying blood concentrations were produced to alter the spectral absorption of Cherenkov light emitted in the tissue. Aqueous solutions of 1% intralipid porcine blood ranging from 0.5% to 3.5% in steps of 0.5% were placed in an array of Petri dishes for simultaneous irradiation. All phantoms were irradiated with 6 MV X-rays at 600 MU min^−1^ from a linear accelerator (Clinac 2100CD, Varian Medical Systems, Palo Alto, CA, USA) with an open 30 × 30 cm^2^ field. Images were acquired at 20 frames per second from the tripod-mounted color Cherenkov camera using a pulse duration of 4 µs and a frame exposure time of 51 ms. All phantom images were acquired with the room lights turned off inside the treatment bunker.

### Patient imaging

Three whole breast radiotherapy patients were imaged as part of an IRB-approved clinical study, with patient consent obtained for acquisition and use of the images. All three patients were being treated supine on the right side, and therefore the color Cherenkov camera was mounted on a tripod and placed against the left wall of the treatment room, preventing gantry occlusion by the left anterior oblique and right posterior oblique tangent fields. Each patient was treated with 6 MV X-rays, and imaging settings were identical to those used for phantom imaging. Treatment room lighting conditions were not changed from the standard lighting levels used, and background subtraction was not performed.

## Supplementary information

Graphical Abstract

Supplementary Material: Figure S1

## Data Availability

All data needed to evaluate the conclusions in this study are present in the paper. Additional data related to this paper may be requested from the authors due to the sensitive nature of patient data.
