# Supplementary material for: Color Cherenkov imaging of clinical radiation therapy
Source: Light Sci Appl. 2021 Nov 4;10:226. doi: 10.1038/s41377-021-00660-0 (PMC8569159; doi:10.1038/s41377-021-00660-0)
Supplement: Supplementary file 1 — Graphical Abstract [file 41377_2021_660_MOESM1_ESM.docx]

Graphical Abstract for “Color Cherenkov Imaging of Clinical Radiation Therapy”

**Short summary:**

A novel three-channel intensified camera capable of imaging Cherenkov emission in full color was developed, allowing for sensitivity to in vivo biological tissue information during radiotherapy.


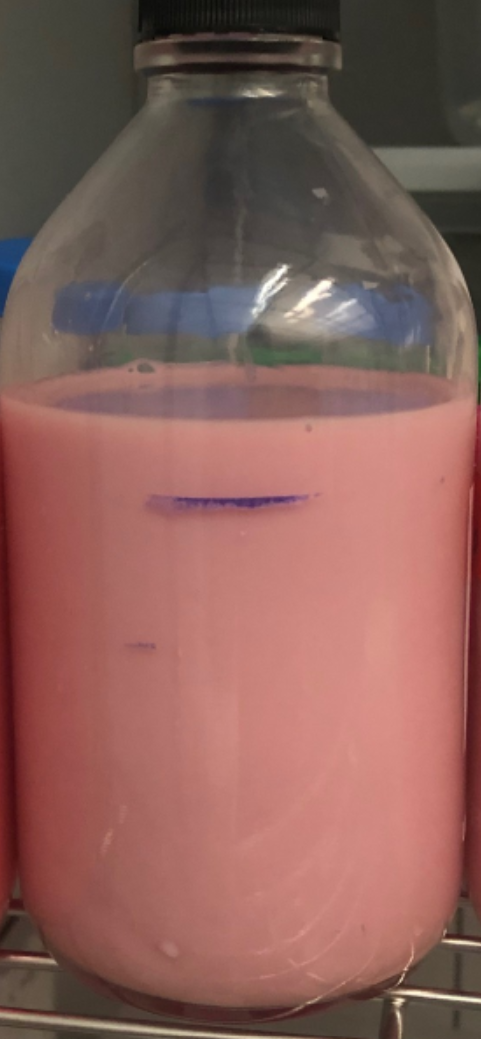

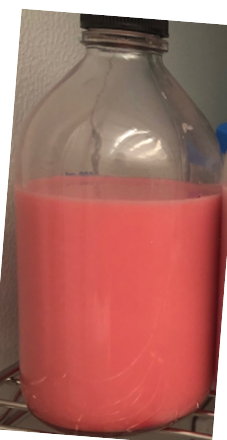

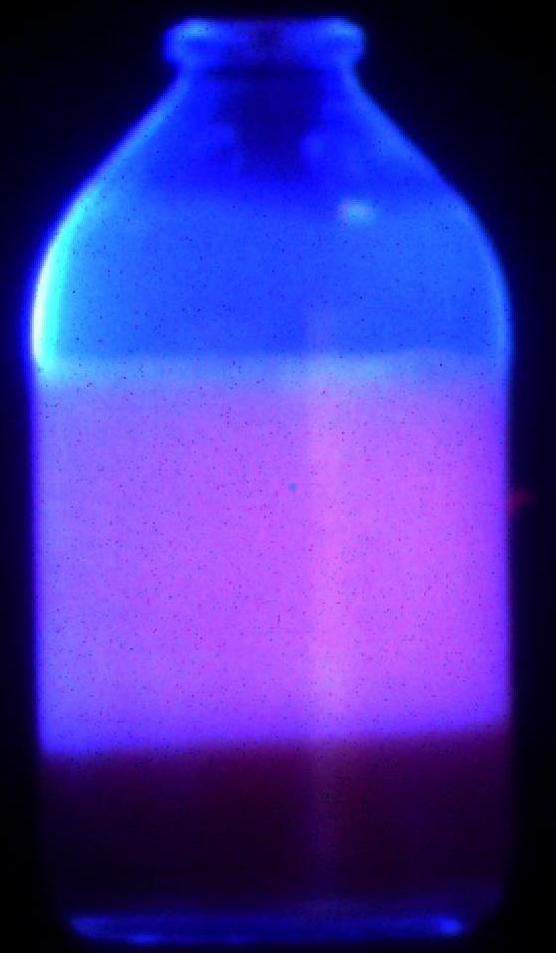

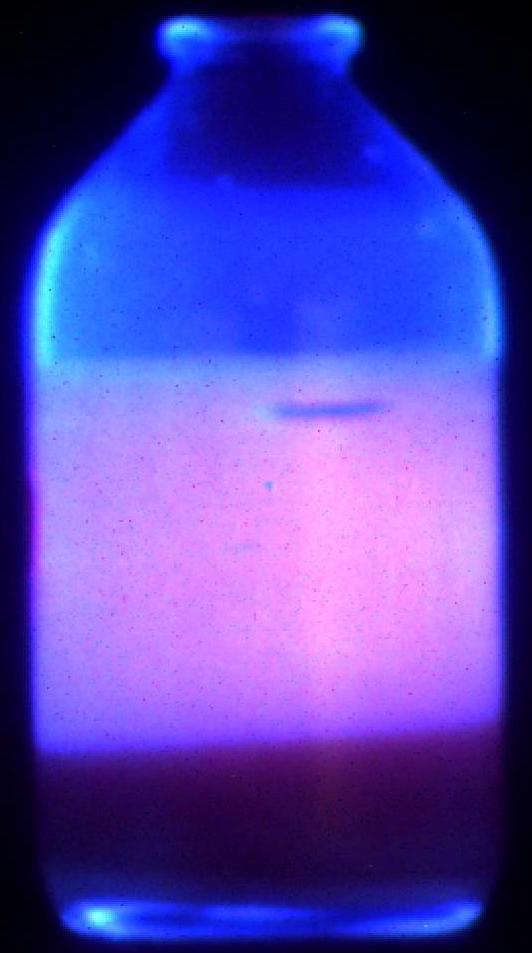

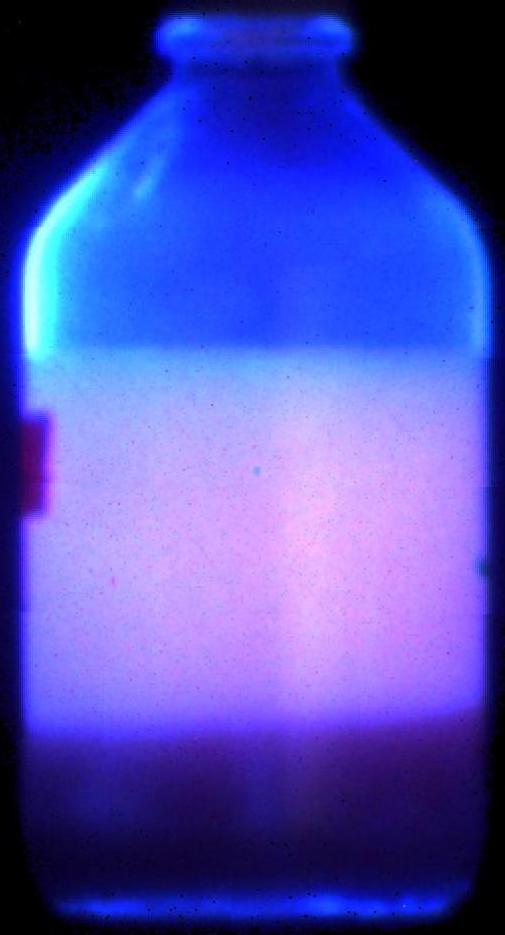


0.5 % Blood

1 % Intralipid

1 % Blood

1 % Intralipid

2 % Blood

1 % Intralipid


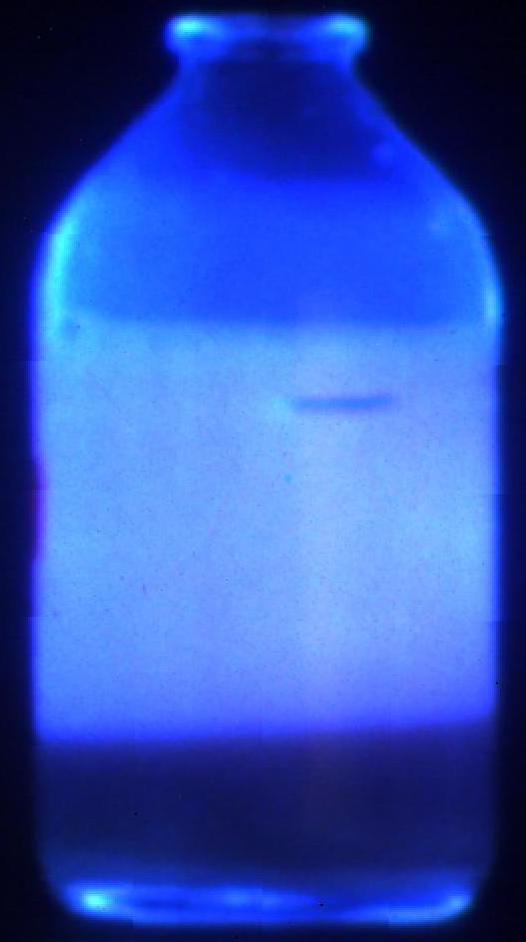

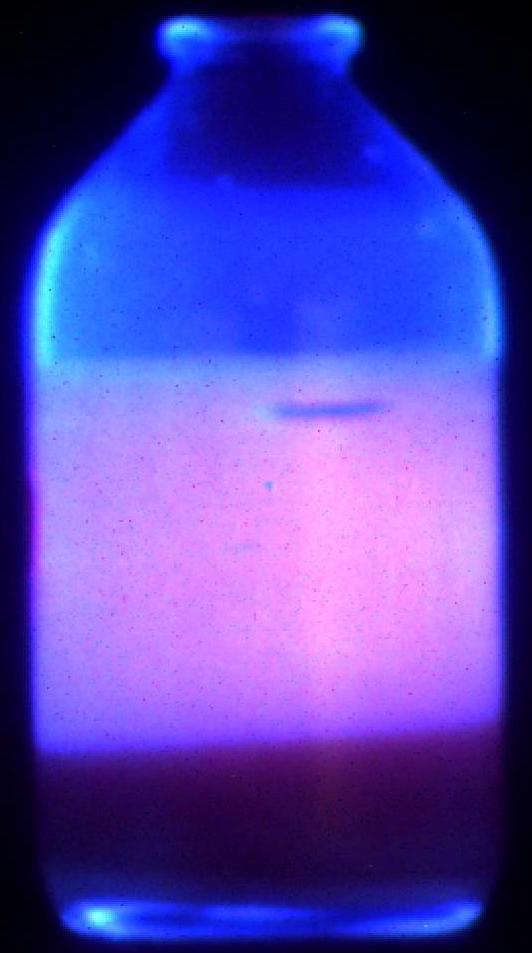


1 % Blood, 1 % Intralipid

**Deoxygenated**

1 % Blood, 1 % Intralipid

**Oxygenated**


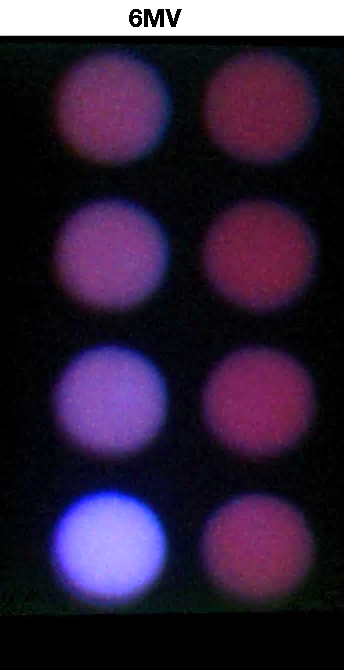


0 %

2 %

0.5 %

2.5 %

1 %

3 %

1.5 %

3.5 %

0.5%

1%

1.5%

3.5%

3%

2.5%


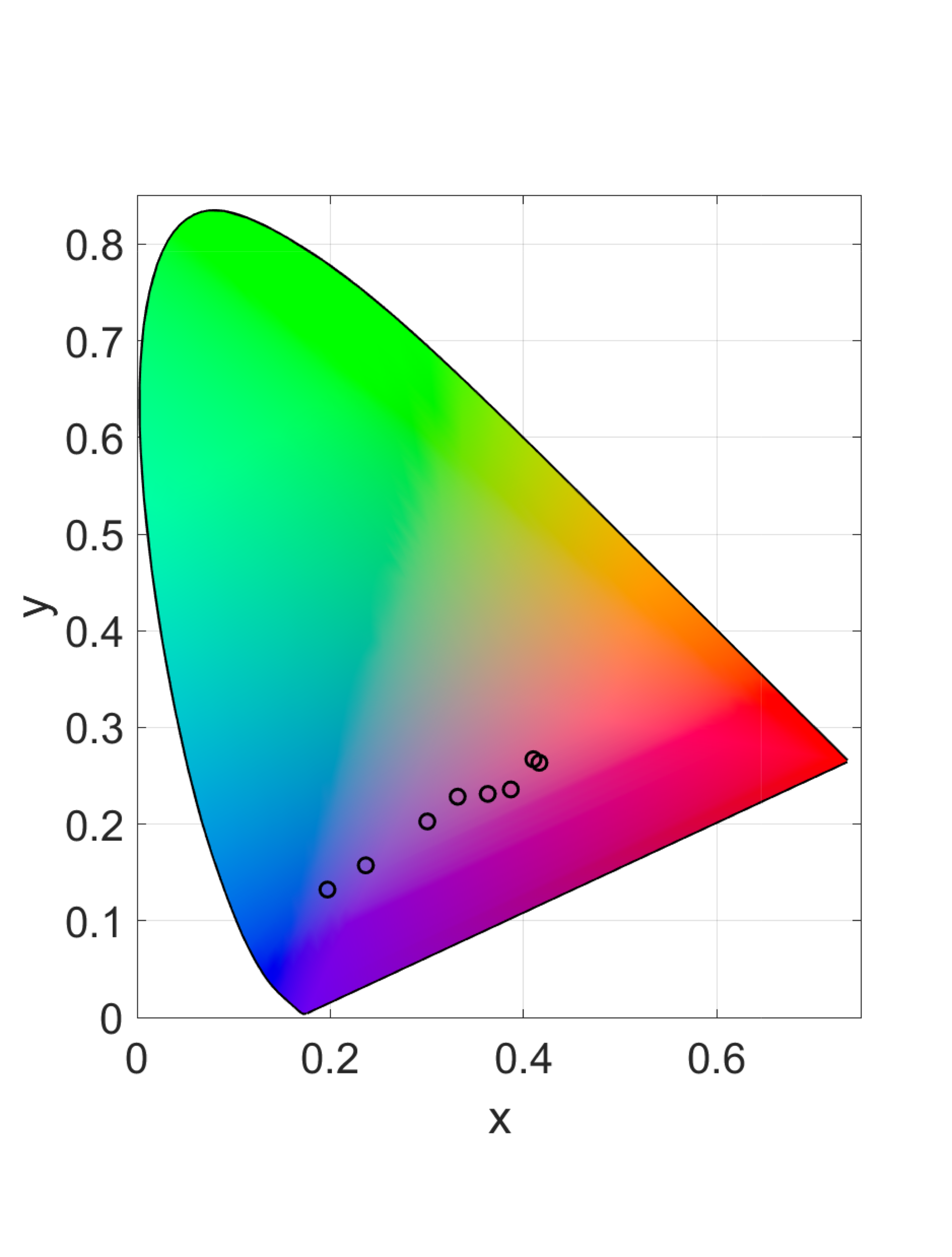

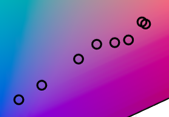


A

B

C

D

0 %

0.5 %

1 %

1.5 %

2 %

3.5 %

2.5 %

3 %
