# Supplementary material for: Color Cherenkov imaging of clinical radiation therapy
Source: Light Sci Appl. 2021 Nov 4;10:226. doi: 10.1038/s41377-021-00660-0 (PMC8569159; doi:10.1038/s41377-021-00660-0)
Supplement: Supplementary file 2 — Supplementary Material: Figure S1 [file 41377_2021_660_MOESM2_ESM.docx]

Supplementary Information

**Color Cherenkov Imaging of Clinical Radiation Therapy**

Daniel A. Alexander^1,2¥^, Anthony Nomezine^1¥^, Lesley A. Jarvis^3,4^, David J. Gladstone^1,3,4^, Brian W. Pogue^1,2,3,4^, Petr Bruza^1,2 *^

^1^Thayer School of Engineering, Dartmouth College, Hanover NH USA

^2^DoseOptics LLC, Lebanon NH USA

^3^Geisel School of Medicine, Dartmouth College, Hanover NH USA

^4^Norris Cotton Cancer Center, Dartmouth-Hitchcock Medical Center, Lebanon NH USA

*Corresponding author. email: [Petr.Bruza@dartmouth.edu](mailto:Petr.Bruza@dartmouth.edu); phone: (603) 667-1406

^¥^equal contribution


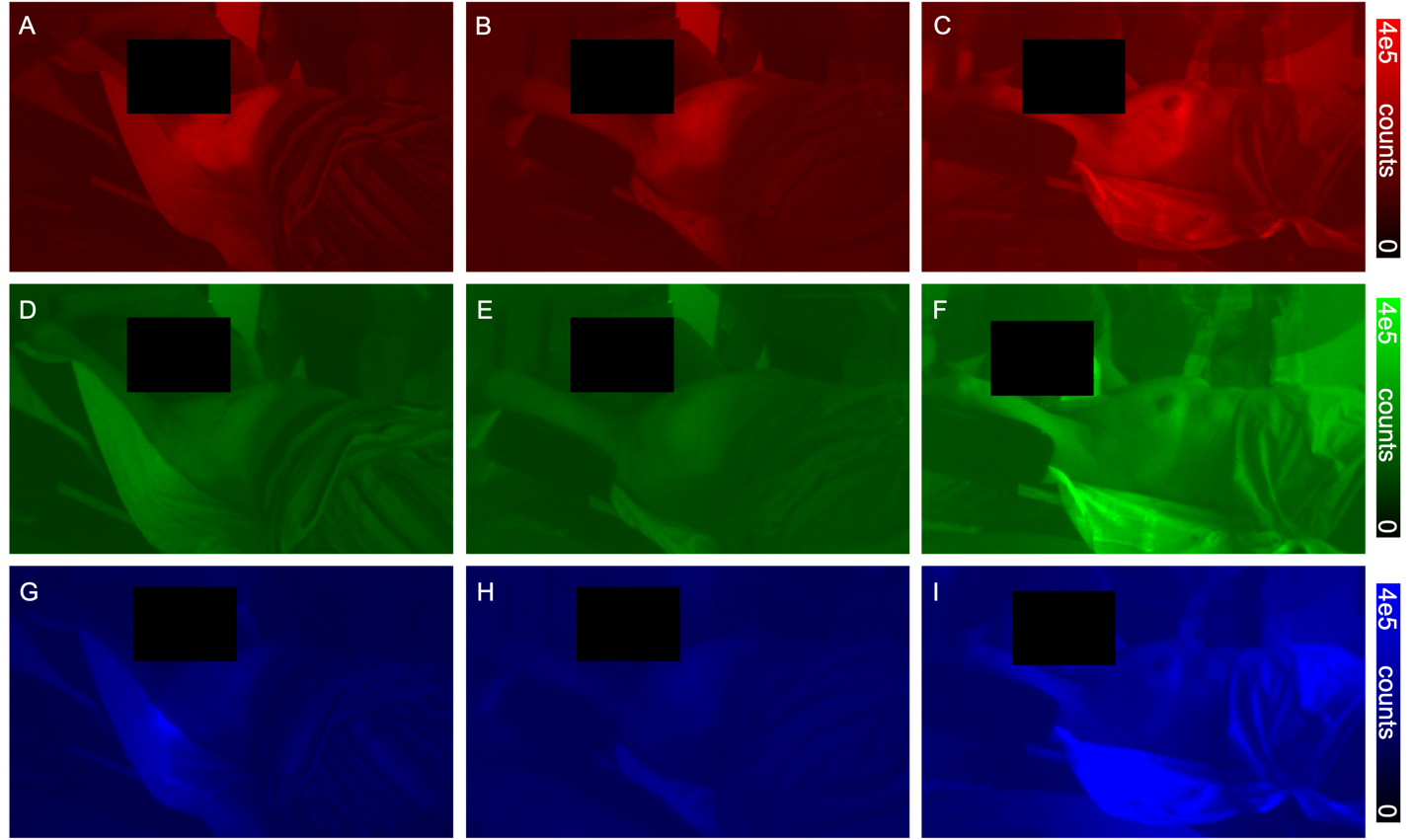


**Figure S1. Raw Three-Channel Cherenkov Images.** Monochromatic raw images from the red, green, and blue channels are shown for the patient images in Figure 4 pre-color correction. The green (D,E,F) and blue (G,H,I) channels display lower signal in the beam area in those wavelength regions, which is expected given the emission spectra in Figure 2.
